# TUC338 Promotes Diffuse Large B Cell Lymphoma Growth via Regulating EGFR/PI3K/AKT Signaling Pathway

**DOI:** 10.1155/2021/5593720

**Published:** 2021-04-19

**Authors:** Yan Li, Zhenwei Jia, Hongbo Zhao, Xiaoyan Liu, Jianmin Luo, Guirong Cui, Xiaoyang Kong

**Affiliations:** Department of Hematology, Handan First Hospital, Handan, Hebei 056002, China

## Abstract

TUC338 is emerging as a novel vital long noncoding RNA (lncRNA) in human cancer; however, its role in diffuse large B cell lymphoma (DLBCL) remains unknown. In this study, we found that TUC338 was remarkably upregulated in DLBCL tissues as compared to matched normal tissues. High TUC338 was closely related to advanced Ann Arbor stage, resistance to CHOP-like treatment, and high IPI (International Prognostic Index). Stable knockdown of TUC338 evidently inhibited cell proliferation and chemotherapy resistance to Adriamycin and induced apoptosis. Further, we found that TUC338 was able to directly bind to miR-28-5p and increased EGFR level, resulting in activating carcinogenic PI3K/AKT signaling, thereby facilitating DLBCL uncontrolled growth. Moreover, we also found that depletion of TUC338 led to the inactivation of EGFR/PI3K/AKT pathway *in vivo* by using the xenograft tumor model. Preclinically, DLBCL patients with high TUC338 had shorter survival time than those with low TUC338, and serum TUC338 level was identified as an excellent indicator for DLBCL diagnosis. In sum, our findings clearly indicate that TUC338 functions as an oncogenic lncRNA in DLBCL through activating EGFR/PI3K/AKT pathway via sponging and inhibiting miR-28-5p, which may be a promising target for DLBCL treatment.

## 1. Introduction

Diffuse large B cell lymphoma (DLBCL) is a kind of malignant tumor originated from B lymphocyte with large heterogeneity and diffuse growth, accounting for 1/3 of the cases of non-Hodgkin's lymphoma [1]. The main clinical symptoms were painless progressive mass enlargement, accompanied by fever and night sweats [2]. The main treatment regimen for DLBCL is systemic chemotherapy, and the first-line chemotherapy regimen is CHOP (cyclophosphamide/doxorubicin/vincristine/prednisone) [3]. The five-year survival rate of DLBCL is very low, because more than half of the patients develop chemotherapy resistance, relapse, and metastasis [4]. Therefore, it is necessary to explore new diagnostic and therapeutic targets to improve patient survival time.

In the field of life science, breakthroughs in noncoding RNA (ncRNA) research in the past 20 years have greatly changed and supplemented the central law. Long noncoding RNA (lncRNA) is an endogenous ncRNA with a length of more than 200 bp [5]. Initially, it was considered as a byproduct of transcription, but with the development of high-throughput sequencing and in-depth research, it has been found that lncRNA plays an indispensable role in biological reproduction, growth and development, aging, and other normal physiological processes, participating in the regulation of the occurrence and development of various diseases, including cancer [6–8]. Functionally, lncRNA is able to effectively sponge miRNA to reduce the suppressive role of miRNA on its target genes, resulting in indirectly increasing gene expression, which is also known as competing endogenous RNA (ceRNA) regulation mechanism [9].

The ceRNA network is frequently deregulated in human cancer, which is involved in cancer occurrence, development, and progression [10]. For example, lncRNA BCYRN1 functioned as a tumor suppressor in glioma by sponging miR-619-5p and elevating CUEDC2, thereby inactivating PTEN/AKT/*p*21 pathway [11]. LncRNA UCA1 was reported as a driver of Warburg effect and carcinogenesis that sponged and inhibited miR-203 activity, leading to increasing HK2 expression in esophageal cancer [12]. Besides, lncRNA PENG could abundantly absorb miR-15b and upregulate PDZK1 level, thus suppressing clear cell renal cell carcinoma cell proliferation [13]. However, little is known about the specific role of ceRNA network in human DLBCL.

Recently, TUC338 has been identified as a key player in tumorigenesis [14–18]. Nevertheless, its function and molecular mechanism in DLBCL remain unexplored. In the present study, we filled this gap and found that TUC338 was significantly upregulated in DLBCL and promoted DLBCL growth by activating EGFR/PI3K/AKT pathway via serving as a ceRNA.

## 2. Materials and Methods

### 2.1. Patients and Specimens

A total of 102 paired DLBCL and normal tissues were obtained from Handan First Hospital. All participants provided written informed consent forms for participating in the study. None of them did receive any anti-tumor treatment and the clinicopathological characteristics are displayed in Table 1. In addition, the serum specimens of 35 DLBCL patients and 35 healthy controls were collected for testing the diagnostic value of TUC338. Our study was approved by the Ethics Committee of Handan First Hospital.

### 2.2. Cell Culture, Transient, and Stable Transfection

Two DLBCL cell lines U2932 and OCI-Ly3 were purchased from ATCC and cultured in DMEM medium supplemented with 10% FBS. Cell transfection was conducted by using Lipofectamine 2000 (Invitrogen, CA, USA). For constructing the cell lines stably silencing TUC338, two validated shRNAs targeting TUC338 were inserted into psi-LVRU6GP lentiviral vector (GeneCopoeia, CA, USA), followed by transfection into U2932 and OCI-Ly3 cells. After 48 h, puromycin was added to culture medium to screen stable cell lines.

### 2.3. RNA Isolation and qRT-PCR

Total RNA was extracted by using TRIZOL reagent according to supplier's instructions (Invitrogen). Besides, PARIS kit (Invitrogen) was used to separate cytoplasmic and nuclear RNA; U6 and GAPDH were applied as control references for nuclear and cytoplasmic fragments, respectively. RNA reverse transcription was conducted by using SuperMix Reverse Transcription Kit (Vazyme Biotech, Nanjing, China), and cDNA amplification and quantification were carried out using SYBR Premix Ex Taq II Kit (Vazyme Biotech).

### 2.4. Cell Proliferation Assay

For soft agar colony formation, 25000 cells were combined with 0.3% agar and then seeded on 0.5% agar layer. Three weeks later, colonies were photographed using an inverted microscope. DNA synthesis rate was assessed by using Cell Proliferation EdU Image Kit (Abbkine, CA, USA) as per supplier's instructions. For CCK-8 assay, the ready-to-use CCK-8 solution was purchased from Abbkine, added to cells, and cultured for 24 h, 48 h, and 72 h. Afterwards, cells were incubated at 37°C for an additional 3 h, and the luminescent data were collected on the EnVision Plate Reader (Perkin Elmer, MA, USA).

### 2.5. RNA Pull-Down Assay

The biotin-labeled TUC338 and anti-sense DNA probes were designed and synthesized by Sangon (Shanghai, China). Afterwards, the probes were added to cell lysates and incubated overnight at 4°C. The streptavidin-magnetic C1 beads (Invitrogen) were added to cell lysates. After washing, the enriched RNA was extracted by TRIZOL, followed by qRT-PCR analysis.

### 2.6. Dual-Luciferase Reporter Gene Assay

The sequence of TUC38 with wild-type or mutant miR-28-5p binding site was synthesized and inserted into pmirGLO reporter (Promega, WI, USA). Then, cells were transfected with miR-28-5p mimics and wild-type or mutant pmirGLO reporter by using Lipofectamine 2000 (Invitrogen). The transfection efficiency was detected by qRT-PCR assay. After 48 h, the luciferase activity in each well was measured using the Dual-Luciferase Reporter System based on supplier's instructions (Promega).

### 2.7. Immunoblotting

Cells were seeded in a 6-well plate and cultured to suitable density. RIPA lysis buffer supplemented with protease inhibitor cocktail tablet and phosphatase inhibitors was added to extract protein. After centrifugation, the supernatant was collected and loaded onto 10% SDS-PAGE, and electro-transferred to a nitrocellulose membrane. After blocking with 5% skim milk powder, the primary antibody was added and incubated overnight at 4°C. The next day, horseradish peroxidase-conjugated secondary antibody was added and incubated for 1h at room temperature. The Novex^®^ Enhanced Chemiluminescence Horseradish Peroxidase Chemiluminescent Substrate Reagent Kit (Invitrogen) was used for further band visualization. The primary antibodies used in this study are as follows: anti-EGFR (#ab32077, Abcam), anti-p-PI3K (#17366, CST), anti-PI3K (#4255, Abcam), anti-p-AKT (#4060, CST), anti-AKT (#4691, CST), and anti-Tubulin (#2148, CST).

### 2.8. Animal Study

The xenotransplantation experiment was carried out using BALB/*c* nude mice provided by GemPharmatech (Nanjing, China). 1 × 10^7^ U2932 cells were injected subcutaneously into nude mice, which were then divided into two groups (sh-NC and sh-TUC338#1; *n* = 5 per group). The mice were grown in the SPF room for 5 weeks; then, all mice were euthanized and tumor tissues were collected and weighed. The animal study was approved by the Animal Care Committee of Handan First Hospital.

### 2.9. Immunohistochemistry (IHC)

Tumor tissues from nude mice were embedded in paraffin and made into paraffin blocks for IHC staining. After dewaxing using xylene and dehydration using gradient concentration ethanol, antigen retrieval was conducted with sodium citrate buffer (pH = 6.0). Then, the section was blocked with 3% H_2_O_2_ and incubated with anti-Ki-67 (#9449, CST), anti-EGFR (#ab32077, Abcam) and anti-p-AKT (#4060, CST). Afterwards, the signaling was visualized by using DAB solution (FineTest, Wuhan, China).

### 2.10. Statistical Analysis

All data were presented as mean ± standard deviation. The relationship between TUC338 expression and clinical data was analyzed by chi-square test. Student's *t*-test was applied to compare the means between the two groups. Kaplan–Meier plotter was used to assess patient's survival, and receiver operating characteristic (ROC) curve analysis was used to measure the diagnostic value of serum TUC338. Two-tailed *P* < 0.05 was considered significant.

## 3. Results

### 3.1. TUC338 Is Significantly Elevated in DLBCL Tissue and Serum Samples

**Figure 1:**
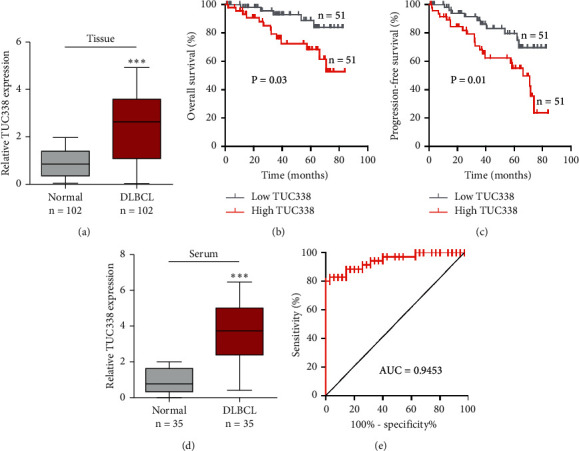
TUC338 is a diagnostic and prognostic biomarker for DLBCL. (a) qRT-PCR analysis of TUC338 expression in 102 paired DLBCL and normal tissues. (b, c). The overall and progression-free survival curves of DLBCL patients with median TUC338 level. (d) qRT-PCR analysis of TUC338 expression in serum from DLBCL patients and healthy controls. (e) ROC curve assessing the diagnostic value of serum TUC338 for DLBCL. ^*∗∗∗*^*P* < 0.001.

As shown in Figure 1(a), TUC338 was notably increased in DLBCL tissues in comparison to matched normal tissues. Then, we analyzed the relationship between TUC338 and clinical features; the results showed that high TUC338 level was closely correlated with advanced Ann Arbor stage, high IPI value, and resistance to CHOP-like treatment. Moreover, patients with high TUC338 had shorter overall as well as progression-free survival than patients with low TUC338 (Figures 1(b) and 1(c)). Importantly, we tested the expression of TUC338 in serum samples; the qRT-PCR assay showed that high TUC338 was also observed in DLBCL serum as compared to healthy serum (Figure 1(d)). And the area under ROC curve (AUC) was 0.9453 (95%CI: 0.8945 to 0.9961) (Figure 1(e)), indicating that serum TUC338 is an excellent diagnostic marker for DLBCL.

### 3.2. TUC338 Knockdown Inhibits DLBCL Cell Malignant Phenotype

**Figure 2:**
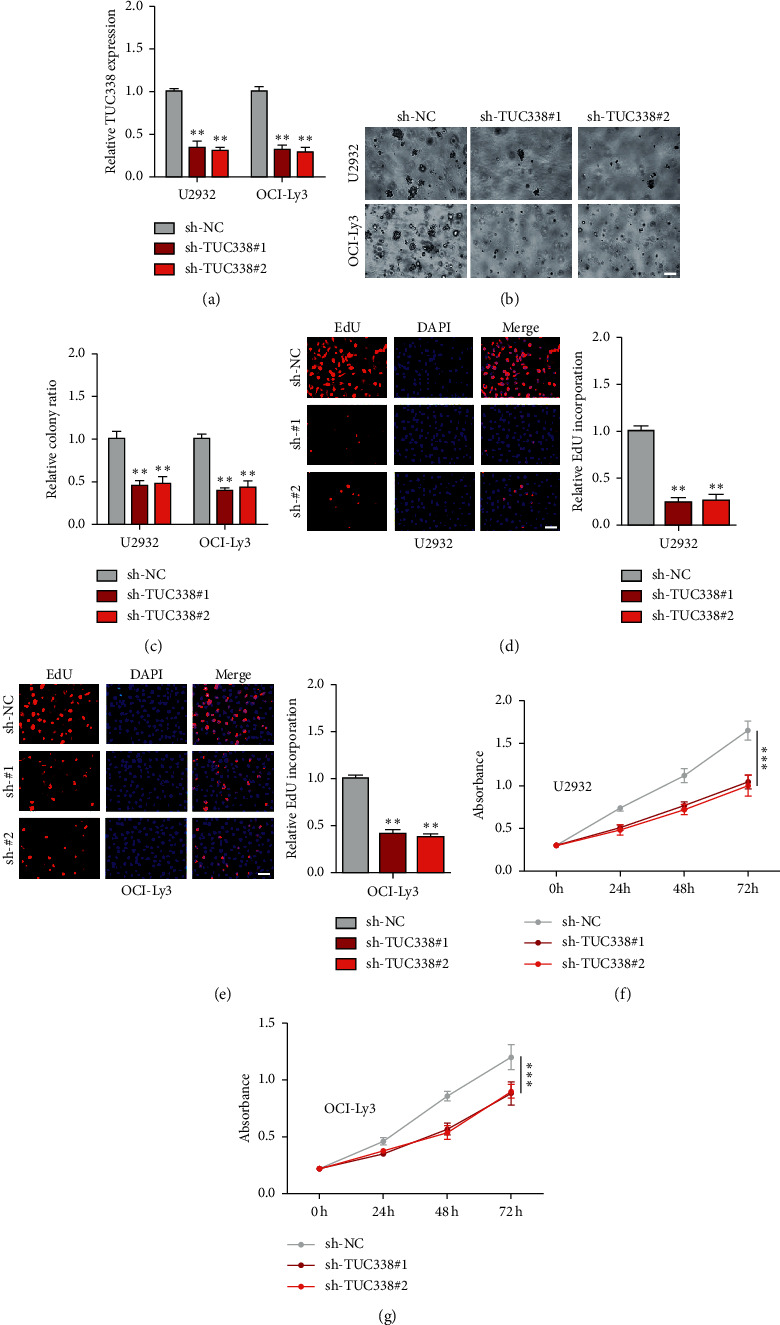
TUC338 knockdown inhibits DLBCL cell proliferation. (a) Establishment of two DLBCL cell lines stably knocking down of TUC338. (b–g) Soft agar colony, EdU, and CCK-8 assays detecting colony formation, DNA synthesis, and cell viability, respectively. Scale bar = 50 *μ*M, ^*∗∗*^*P* < 0.01, ^*∗∗∗*^*P* < 0.001.

To explore the function of TUC338 in DLBCL, we established stable TUC338 knockdown cell lines (Figure 2(a)). As shown in Figures 2(b) and 2(c), inhibition of TUC338 in both U2932 and OCI-Ly3 cells reduced colony formation in soft agar by more than 50% compared with nontargeting control. Besides, the DNA synthesis rate was significantly slowed down after TUC338 knockdown, as shown by EdU staining assay (Figures 2(d) and 2(e)). Likewise, CCK-8 assay showed that depletion of TUC338 resulted in a sharp reduction in cell viability (Figures 2(f) and 2(g)). And flow cytometry showed that more cells underwent apoptosis (Figure S1A, B) and were arrested in the G0/*G*1 phase after TUC338 knockdown (Figure S1C, D). Moreover, we also established Adriamycin-resistant DLBCL cells and found that TUC338 was significantly upregulated in Adriamycin-resistant cells compared to control cells (Figure S1E), and knockdown of TUC338 substantially reduced chemotherapy resistance (Figure S1F).

### 3.3. TUC338 Sponges miR-28-5p in DLBCL Cells

**Figure 3:**
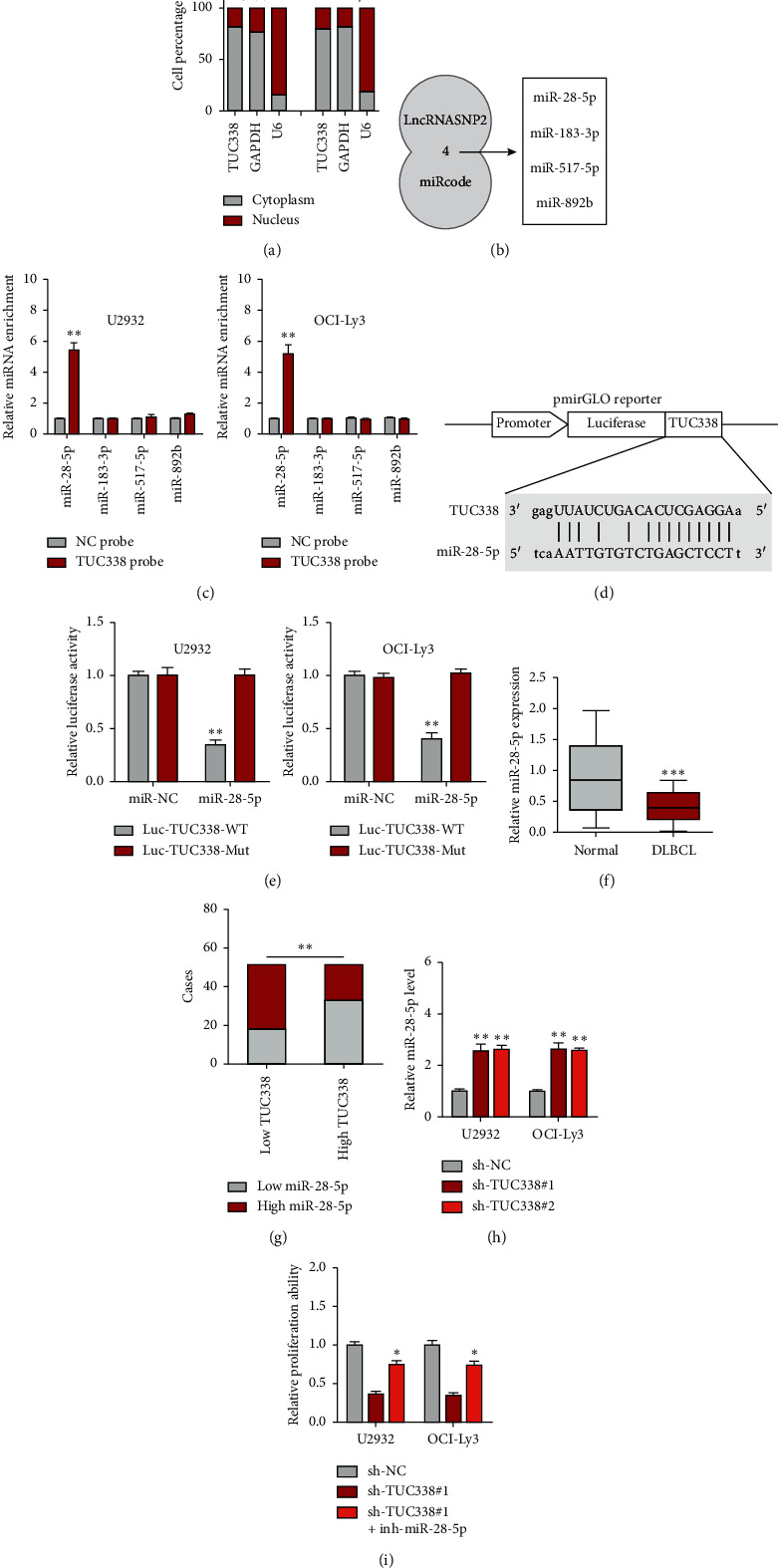
miR-28-5p is sponged by TUC338 in DLBCL cells. (a) qRT-PCR analysis detecting subcellular localization of TUC338 in DLBCL cell lines. (b) Two online tools predicting the miRNAs bound by TUC338. (c) RNA pull-down assay using biotin-labeled probe, followed by qRT-PCR analysis of miRNA enrichment. (d, e) The wild-type or mutant TUC338 sequence was cloned into pmirGLO vector, and luciferase reporter assay was performed in DLBCL cells co-transfected with miR-28-5p mimics and the above pmirGLO vector. (f) qRT-PCR analysis of miR-28-5p expression in paired DLBCL and normal tissues. (g) The correlation analysis between TUC338 and miR-28-5p in DLBCL tissues. (h) qRT-PCR analysis of miR-28-5p expression after TUC338 knockdown. (i) Cell proliferation assay in TUC338-silenced DLBCL cells transfected with miR-28-5p inhibitor. ^*∗*^*P* < 0.05, ^*∗∗*^*P* < 0.01, ^*∗∗∗*^*P* < 0.001.

Then, we analyzed the location of TUC338; the qRT-PCR results showed that it was predominantly located in the cytoplasm in both U2932 and OCI-Ly3 cells (Figure 3(a)). The cytoplasmic lncRNA mainly serves as a miRNA sponge; thus we analyzed the online tools lncRNASNP2 and miRcode; the overlying results showed four miRNAs that may be bound by TUC338 (Figure 3(b)). To verify this prediction, we designed biotinylated TUC338 and its anti-sense control probes to conduct RNA pull-down assay. The results showed that only miR-28-5p was significantly pulled down by TUC338 probe in both U2932 and OCI-Ly3 cells (Figure 3(c)). Then, the TUC338 sequence with wild-type or mutant miR-28-5p binding site was cloned into pmirGLO reporter (Figure 3(d)). As shown in Figure 3(e), miR-28-5p overexpression led to a substantial reduction in the luciferase activity of wild-type vector but had no effect on the luciferase activity of mutated vector (Figure 3(e)). Next, we tested the expression of miR-28-5p in DLBCL tissues; the results showed that its expression was significantly decreased in DLBCL as compared to normal tissues (Figure 3(f)). And a strong negative correlation between TUC338 and miR-28-5p was observed in DLBCL tissues (Figure 3(g)). In addition, miR-28-5p was notably increased in TUC338-depleted U2932 and OCI-Ly3 cells as compared to control cells (Figure 3(h)). Functionally, the reduced cell proliferation induced by TUC338 knockdown was evidently rescued after silencing of miR-28-5p (Figure 3(i)).

### 3.4. TUC338 Regulates EGFR/PI3K/AKT Signaling via miR-28-5p in DLBCL Cells

**Figure 4:**
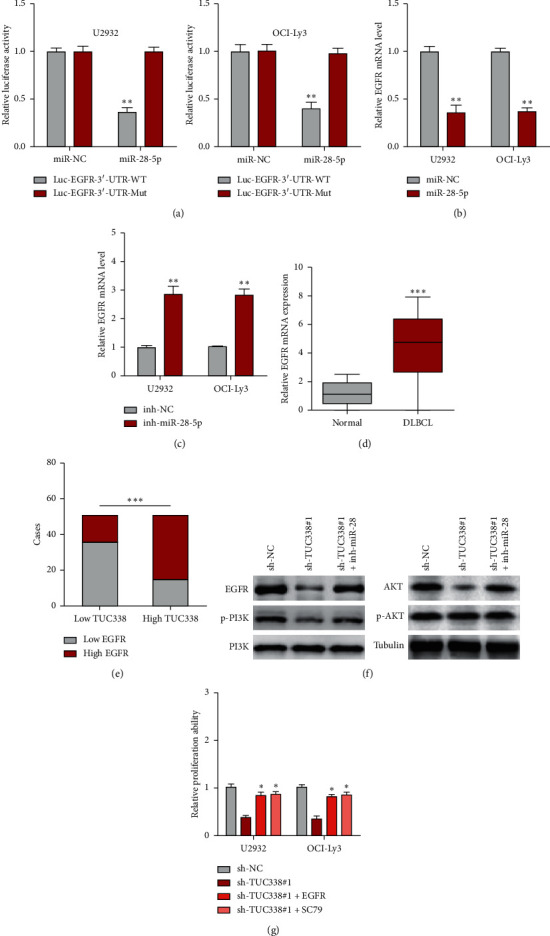
TUC338 activates EGFR/PI3K/AKT pathway via miR-28-5p. (a) luciferase reporter assay in DLBCL cells co-transfected with wild-type or mutant pmirGLO vector and miR-28-5p mimics. (b, c). qRT-PCR analysis of EGFR mRNA expression after miR-28-5p overexpression or silencing. (d) qRT-PCR analysis of EGFR mRNA level in paired DLBCL and normal tissues. (e) The correlation analysis between TUC338 and EGFR in DLBCL tissues. (f) Immunoblotting detecting the indicated protein levels in TUC338-silenced DLBCL cells transfected with miR-28-5p inhibitor. (g) Cell proliferation assay in TUC338-silenced DLBCL cells transfected with EGFR-expressing plasmid or treated with SC79. ^*∗*^*P* < 0.05, ^*∗∗*^*P* < 0.01, ^*∗∗∗*^*P* < 0.001.

By analyzing the experimentally validated TarBase 7.0 database, we found that oncogene EGFR was the direct target of miR-28-5p. Then, luciferase reporter assay was carried out and the results showed that miR-28-5p overexpression significantly reduced the luciferase activity of wild-type EGFR 3`-UTR reporter, but this effect was blocked after mutation of the binding site between miR-28-5p and EGFR 3`-UTR (Figure 4(a)). Likewise, EGFR mRNA was significantly reduced after miR-28-5p overexpression (Figure 4(b)), and silencing of miR-28-5p resulted in an opposite effect (Figure 4(c)). We then tested the level of EGFR in DLBCL tissues, as shown in Figure 4(d); high EGFR was observed in DLBCL as compared to normal tissues, which was positively correlated with TUC338 level (Figure 4(e)). Consistently, EGFR protein expression was evidently decreased in TUC338-silenced DLBCL cells, accompanied by inhibition of its downstream PI3K/AKT signaling pathway, whereas this phenomenon was significantly abolished after silencing of miR-28-5p (Figure 4(f)). Functionally, the attenuated cell proliferation caused by TUC338 knockdown was effectively rescued by EGFR overexpression or treatment with SC79, a pharmacological activator of AKT signaling (Figure 4(g)).

### 3.5. Depletion of TUC338 Retards Tumor Growth *In Vivo*

**Figure 5:**
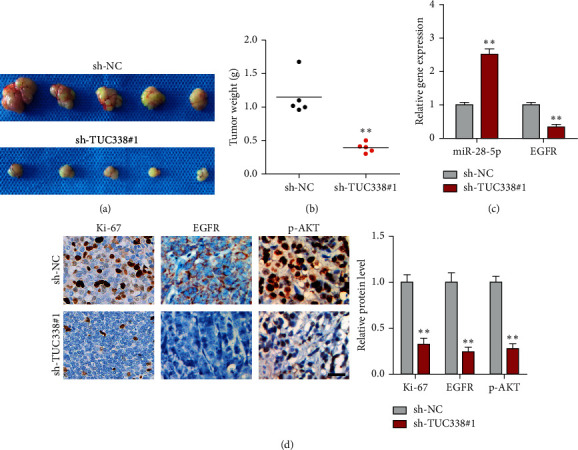
Silencing of TUC338 inhibits *in vivo* tumor growth. (a, b). The image and wight of subcutaneous tumors in control and TUC338-depleted groups. (c) qRT-PCR analysis of EGFR mRNA and miR-28-5p levels in control and TUC338-depleted groups. (d) IHC staining detecting the protein levels of Ki-67, EGFR, and p-AKT in control and TUC338-depleted groups. Scale bar = 50 *μ*M, ^*∗∗*^*P* < 0.01.

Lastly, to test the *in vivo* function of TUC338, we subcutaneously injected control or TUC338-silenced U2932 cells into nude mice. Five weeks later, we found that tumor weight in TUC338 knockdown group was significantly smaller than that in control group (Figure 5(a) and 5(b)). Importantly, miR-28-5p level was increased, while EGFR mRNA level was decreased in mice bearing TUC338-depleted tumors (Figure 5(c)). Moreover, less Ki-67 (proliferation marker), EGFR, and p-AKT positive cells were observed in TUC338-depleted group in comparison to control group (Figure 5(d)).

## 4. Discussion

Despite great advances in diagnosis and treatment of DLBCL over the decades, DLBCL is still an intractable disease with high clinical heterogeneity, recurrence, and mortality, elucidating that the complex pathogenesis remains a top priority. In the current study, we identified TUC338 as a DLBCL-related lncRNA; TUC338 promoted DLBCL cell proliferation and growth both *in vitro* and *in vivo* by activating EGFR/PI3K/AKT signaling pathway. Further, TUC338 was mainly located in the cytoplasm, where it abundantly sponged miR-28-5p and segregated the interaction between miR-28-5p and EGFR 3`-UTR, resulting in EGFR upregulation and subsequently activation of oncogenic PI3K/AKT pathway. Therefore, our data provide a new insight into the pathogenesis of DLBCL, as well as a new experimental basis for the important role of lncRNA in human cancer.

Many diseases are accompanied by changes in certain biochemical indicators in cells or tissues, such as changes in molecular expression. Biochemical indicators that can show changes in the structure or function of systems, organs, tissues, cells, and subcells, or changes that may occur, are called biomarkers [19]. The development and application of biomarkers play an important role in disease diagnosis, disease stage judgment, and new drug development [20]. Emerging evidence suggests that lncRNA is an excellent biomarker for cancer diagnosis and prognosis [21]. For instance, SChLAP1 was a lncRNA specifically expressed in prostate cancer cells, which could be used as an effective biomarker in the identification and postoperative observation of prostate cancer [22]. And by analyzing genome-wide RNA sequencing libraries from 25 independent studies, Iyer et al. found a total of 7942 lncRNA that might act as potential biomarkers for specific cancer tissues [23]. With the deepening of research on lncRNA, more lncRNA will be used in the detection and development of biomarkers, providing effective help for human to defeat cancer. Herein, we found that DLBCL patients with high TUC338 had shorter survival than those with low TUC338, implying that TUC338 is a prognostic biomarker for DLBCL. Furthermore, serum TUC338 was also upregulated in DLBCL patients compared to healthy controls, and the AUC value was 0.9453, indicating that serum TUC338 is an excellent noninvasive diagnostic biomarker for DLBCL. Further study is warranted to validate the diagnostic and prognostic efficacy of TUC338 in multi-center, large-sample, and randomized controlled trials.

The abnormal ceRNA network is closely linked to human cancer. The premise for lncRNA to play the role of ceRNA is that it is located in the cytoplasm, where it can directly bind to cytoplasmic mature miRNAs loaded into RNA-induced silencing complex (RISC) by AGO2 [24]. In this study, we identified TUC338 as a cytoplasmic lncRNA in DLBCL cells that could sponge miR-28-5p and inhibit its activity. miR-28-5p is a well-documented tumor suppressor in various cancer types, including breast cancer [25], pancreatic cancer [26, 27], endometrial cancer [28], colorectal cancer [29], and hepatocellular carcinoma [30]. Here, we found that miR-28-5p level was significantly decreased in human DLBCL tissues, and silencing of miR-28-5p could effectively block the decreased cell malignant phenotype caused by TUC338 knockdown, suggesting that miR-28-5p also plays a tumor-inhibiting role in DLBCL. Further, we found that the well-known oncogene EGFR was the direct target of miR-28-5p. EGFR, also named as HER1 or ERBB1, is a transmembrane tyrosine kinase receptor that binds to the extracellular ligand to form a dimer and phosphorylates the intracellular domain, subsequently phosphorylating and activating PI3K, resulting in the activation of PI3K/AKT signaling [31]. TUC338 elevated EGFR level and activated PI3K/AKT pathway by sponging and inhibiting miR-28-5p. And overexpression of EGFR or treatment with AKT signaling activator significantly rescued the attenuated cell proliferation induced by TUC338 depletion, indicating that the ceRNA regulatory axis of TUC338/miR-28-5p/EGFR/PI3K/AKT does exist in DLBCL cells.

In summary, our data for the first time reveal the protumor role of TUC338 in DLBCL; the deregulated ceRNA network of TUC338/miR-28-5p/EGFR/PI3K/AKT may be responsible for DLBCL tumorigenesis. TUC338 can be utilized as a biomarker for the diagnosis and prognosis of DLBCL as well as a therapeutic target.

## Figures and Tables

**Table 1:** Association of TUC338 expression with clinical parameters in DLBCL patients (*n* = 102).

Parameters	Total (*n* = 102)	TUC338 expression		*P* value
		Low (*n* = 51)	High (*n* = 51)	
Age				0.692
≤60	48	23	25	
>60	54	28	26	
Gender				0.313
Male	41	18	23	
Female	61	33	28	
Ann Arbor stage				0.016
I-II	44	28	16	
III-IV	58	23	35	
CHOP-like treatment				<0.001
Response	52	40	12	
Nonresponse	50	11	39	
IPI				<0.001
0-2	37	28	9	
3-5	65	23	42	

## Data Availability

The datasets used and analyzed during the current study are available from the corresponding author on reasonable request.

## Supplementary Materials

Supplementary Materials Figure S1: knockdown of TUC338 promotes apoptosis and inhibits cell cycle progression and chemotherapy resistance. (A, B) Flow cytometry analyzing the number of apoptotic U2932 cells after TUC338 silencing. (C, D) Flow cytometry analyzing the cell percentage in different phases after TUC338 silencing. (E) qRT-PCR analysis of TUC338 in Adriamycin-resistant U2932 cells. (F) Flow cytometry analyzing the number of apoptotic Adriamycin-resistant U2932 cells after TUC338 silencing. ^*∗∗*^*P* < 0.01.
