# Supplementary material for: TUC338 Promotes Diffuse Large B Cell Lymphoma Growth via Regulating EGFR/PI3K/AKT Signaling Pathway
Source: J Oncol. 2021 Apr 19;2021:5593720. doi: 10.1155/2021/5593720 (PMC8079195; doi:10.1155/2021/5593720)
Supplement: Supplementary Materials — Figure S1: knockdown of TUC338 promotes apoptosis and inhibits cell cycle progression and chemotherapy resistance. (A, B) Flow cytometry analyzing the number of apoptotic U2932 cells after TUC338 silencing. (C, D) Flow cytometry analyzing the cell percentage in different phases after TUC338 silencing. (E) qRT-PCR analysis of TUC338 in Adriamycin-resistant U2932 cells. (F) Flow cytometry analyzing the number of apoptotic Adriamycin-resistant U2932 cells after TUC338 silencing. ∗∗P < 0.01. [file 5593720.f1.doc]

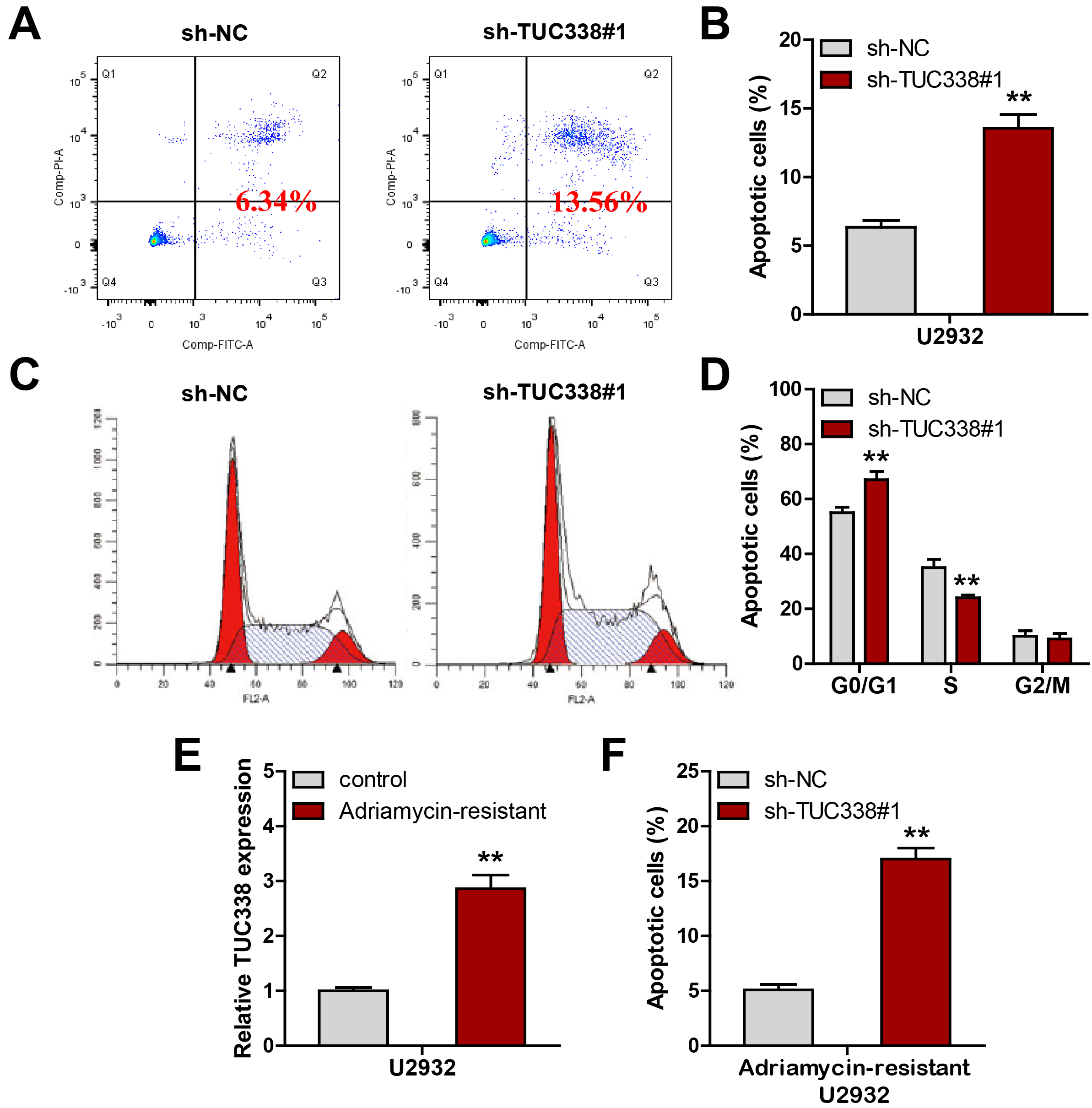


**Figure S1:** Knockdown of TUC338 promotes apoptosis, inhibits cell cycle progression and chemotherapy resistance. A, B. Flow cytometry analyzing the number of apoptotic U2932 cells after TUC338 silencing. C, D. Flow cytometry analyzing the cell percentage in different phases after TUC338 silencing. E. qRT-PCR analysis of TUC338 in Adriamycin-resistant U2932 cells. F. Flow cytometry analyzing the number of apoptotic Adriamycin-resistant U2932 cells after TUC338 silencing. **^**^***P*<0.01.
